# Combination Testing Using a Single *MSH5* Variant alongside HLA Haplotypes Improves the Sensitivity of Predicting Coeliac Disease Risk in the Polish Population

**DOI:** 10.1371/journal.pone.0139197

**Published:** 2015-09-25

**Authors:** Agnieszka Paziewska, Bozena Cukrowska, Michalina Dabrowska, Krzysztof Goryca, Magdalena Piatkowska, Anna Kluska, Michal Mikula, Jakub Karczmarski, Beata Oralewska, Anna Rybak, Jerzy Socha, Aneta Balabas, Natalia Zeber-Lubecka, Filip Ambrozkiewicz, Ewa Konopka, Ilona Trojanowska, Malgorzata Zagroba, Malgorzata Szperl, Jerzy Ostrowski

**Affiliations:** 1 Department of Gastroenterology Hepatology and Clinical Oncology, Medical Center for Postgraduate Education, Warsaw, Poland; 2 Department of Pathology, The Children’s Memorial Health Institute, Warsaw, Poland; 3 Department of Genetics, Maria Sklodowska-Curie Memorial Cancer Center and Institute of Oncology, Warsaw, Poland; 4 Department of Gastroenerology, Hepatology, Nutritional Disorders and Pediatrics, The Children’s Memorial Health Institute, Warsaw, Poland; 5 The Faculty of Health Protection and Human Sciences, the State Higher School of Vocational Education, Ciechanow, Poland; 6 Department of Molecular Biology, The Cardinal Stefan Wyszynski Institute of Cardiology, Warsaw, Poland; Hospital Israelita Albert Einstein, BRAZIL

## Abstract

Assessment of non-HLA variants alongside standard HLA testing was previously shown to improve the identification of potential coeliac disease (CD) patients. We intended to identify new genetic variants associated with CD in the Polish population that would improve CD risk prediction when used alongside HLA haplotype analysis. DNA samples of 336 CD and 264 unrelated healthy controls were used to create DNA pools for a genome wide association study (GWAS). GWAS findings were validated with individual HLA tag single nucleotide polymorphism (SNP) typing of 473 patients and 714 healthy controls. Association analysis using four HLA-tagging SNPs showed that, as was found in other populations, positive predicting genotypes (*HLA-DQ2*.*5/DQ2*.*5*, *HLA-DQ2*.*5/DQ2*.*2*, and *HLA-DQ2*.*5/DQ8*) were found at higher frequencies in CD patients than in healthy control individuals in the Polish population. Both CD-associated SNPs discovered by GWAS were found in the CD susceptibility region, confirming the previously-determined association of the major histocompatibility (MHC) region with CD pathogenesis. The two most significant SNPs from the GWAS were rs9272346 (HLA-dependent; localized within 1 Kb of *DQA1*) and rs3130484 (HLA-independent; mapped to *MSH5*). Specificity of CD prediction using the four HLA-tagging SNPs achieved 92.9%, but sensitivity was only 45.5%. However, when a testing combination of the HLA-tagging SNPs and the *MSH5* SNP was used, specificity decreased to 80%, and sensitivity increased to 74%. This study confirmed that improvement of CD risk prediction sensitivity could be achieved by including non-HLA SNPs alongside HLA SNPs in genetic testing.

## Introduction

Coeliac disease (CD) is a chronic immune-mediated enteropathy that affects approximately 1% of European individuals [1]. CD is triggered by exposure to dietary gluten in genetically predisposed individuals [2]. Serological screening studies showed that the majority of CD cases go unrecognized. Lack of diagnosis and the consequent lack of appropriate treatment (strict gluten-free diet) can lead to treatment-resistant CD, malignancy (especially T-cell lymphoma), and other autoimmune disorders [3,4]. Individuals with pre-existing immune-mediated diseases (e.g., diabetes mellitus type 1 (DMT1), autoimmune hepatitis, and thyroiditis) and certain genetic diseases (e.g., Down syndrome, Turner syndrome, and William syndrome) are at high risk of developing CD, as are members of families that contain CD patients [5–10].

Almost all patients with CD carry HLA-DQ2.5 heterodimers encoded by DQA1*05 and DQB1*02 alleles, both in cis or in trans configuration, along with HLA-DQ8, encoded by DQB1*03:02 generally in combination with the DQA1*03 variant [11]. Consequently, according to the current criteria of the European Society for Gastroenterology, Hepatology and Nutrition (ESPGHAN) [12], CD can be diagnosed, without the need for additional documentation of characteristic histological findings on duodenal biopsy, in symptomatic patients who exhibit high levels of anti-tissue transglutaminase 2 (tTg2)-IgA antibodies in repeated testing and who also carry HLA risk haplotypes. CD screening at an early age may be advisable for patients at high risk of CD development and children with the HLA *DQ2* haplotype (particularly homozygous individuals) [11].

Although the negative predictive value of specific HLA alleles reaches almost 100%, the positive predictive value of HLA risk haplotypes is much less informative as these are found in approximately 40% of the general population [13]. A recent study, which added 57 non-HLA single-nucleotide polymorphisms (SNPs) to the prediction model, improved the discriminatory power of genetic testing and facilitated the reclassification of individuals into more accurate CD risk categories [14].

The aim of the present study was to identify novel genetic variants associated with CD in the Polish population that could be used alongside HLA haplotypes to improve CD risk prediction. A pooled-DNA allelotyping genome wide association study (GWAS) was performed to identify putative informative variants. This was followed by PCR-based genotyping of individual DNA samples. The GWAS uncovered several SNPs on 6p21.3 that were associated with CD. Association with CD had not been reported prior to this study for one of the highly significant non-HLA SNPs, rs3130484, which mapped to *MSH5*. This single *MSH5* variant significantly improved the sensitivity of CD risk prediction when used alongside HLA haplotyping.

## Materials and Methods

### Ethics statement

All enrolled patients and control subjects were Polish Caucasians. The study was approved by the ethics committee of the Children’s Memorial Health Institute, Warsaw, Poland, and all participants or their parents provided written informed consent. The study protocol conforms to the ethical guidelines of the 1975 Declaration of Helsinki.

### Studied subjects

The GWAS cohort comprised 336 CD patients (216 females and 120 males), 255 of who were diagnosed with CD before 14 years of age, and 264 unrelated healthy individuals (213 females and 51 males) who tested negative for anti-tTg2-IgA and anti-deamidated gliadin peptide (DGP)-IgG antibodies. CD was diagnosed according to ESPGHAN guidelines [12]. The presence of typical histopathological changes at or above grade 2 (modified Marsh–Oberrhuber classification) in duodenal specimens obtained during endoscopy or, in some cases in whom biopsy was not undertaken, very high concentrations of anti-tTg2-IgA or antibodies were considered indicative of CD.

A larger cohort of CD patients and control individuals was enrolled in a replication study. This comprised 473 CD patients (299 females and 174 males), 358 of who were diagnosed before 14 years of age, 357 CD family members (224 females and 133 males) who tested negative for anti-tTg2-IgA/DGP-IgG antibodies, and 714 unrelated healthy controls (334 females and 380 males).

### Allelotyping GWAS

A pooled-DNA sample-based GWAS was performed as described previously [15]. Genomic DNA was extracted from whole blood treated with EDTA using a QIAamp DNA Mini Kit. DNA sample concentrations were measured using a Quant-iT^TM^ PicoGreen dsDNA Kit (Invitrogen, United Kingdom). DNA integrity was verified by 1% agarose gel analysis.

DNA samples that passed quality control tests were combined according to the patient age of diagnosis and sex at equimolar concentrations to obtain pools which consisted of 33 to 44 DNA samples. Pooled-DNA samples were adjusted to a final concentration of 50 ng/ml in Tris-EDTA buffer (pH = 8). Nine and six DNA pools were prepared for CD and control samples, respectively. DNA pools were assayed independently on Affymetrix Genome-Wide Human SNP 6.0 by the external AROS Applied Biotechnology A/S (Aarhus, Denmark) service. GWAS datasets used in the manuscript are available in GEO database under GSE65424.

### Individual genotyping

For the validation of GWAS findings and for the HLA tag SNP typing replication study, individual patients and controls were genotyped with TaqMan SNP Genotyping Assays (Life Technologies, USA) using a SensiMix TM II Probe Kit (Bioline Ltd, United Kingdom) and a 7900HT Real-Time PCR system (Life Technologies, USA).

### Statistical analyses–allelotyping GWAS

The intensity of each SNP was calculated as the relative allele signal (RAS) for each microarray, as described previously [15]. Student’s *t*-test (Welch variant) was used to compare allele ratios between groups. Distribution assumptions were verified by visual inspection of the p-value QQ plot (S1 Fig). Two comparisons were performed: using complete sample sets or after removing one control sample according to higher MAPD (Median of the Absolute value of all Pairwise Differences between log2 ratios for a given chip). No probe filtering was performed. P-values were corrected for multiple hypothesis testing by the Benjami-Hochberg algorithm. Manhattan plotting was performed using the qqman R package [16].

### Statistical analyses–individual genotyping

Associations were examined using chi^2^-tests implemented in R (version 3.1.1). The odds ratios (ORs) and 95% confidence intervals (CIs) were estimated by normal approximation using the EpiTools R package [17]. Conditional logistic regression models were constructed using "clogit" function in R (approximate method).

Predictive models were constructed to maximize sensitivity while achieving at least 80% specificity (without any knowledge of the underlying biological mechanism). When the *MSH5* risk variant and HLA haplotypes were considered together, individuals who carried a single minor allele at the rs3130484 locus or who carried any of the HLA-DQ2.5 risk genotypes (*DQ2*.*5/2*.*5*, *DQ2*.*5/2*.*2*, *or DQ2*.*5/8*) were considered at risk. When the *MSH5* and *HLA-DQA1* risk variants were considered together, individuals carrying a single minor allele at the rs3130484 locus and one minor allele at the rs9272346 locus were considered at risk.

The independence of MSH5 and HLA effects was checked by building a logistic regression model that included MSH5 and HLA terms along the appropriate cross-term. The significance (or lack of) of effect for cross-term was considered a marker of independence.

## Results

### HLA tag SNP typing

DNA samples from 473 CD patients and 715 controls were genotyped using TaqMan SNP Genotyping Assays for four previously identified [18] HLA-tagging SNPs. The positive predicting genotypes *HLA-DQ2*.*5/DQ2*.*5*, *HLA-DQ2*.*5/DQ2*.*2*, and *HLA-DQ2*.*5/DQ8* were found more frequently in the CD patient group than in the control group. This confirmed the increased CD risk associated with these genotypes compared to the *HLA-DQ2*.*2/DQ2*.*2*, *HLA-DQ8/DQ8*, and *HLA-DQ8/DQ2*.*2* genotypes, which did not differ in frequency between the CD and control groups (Table 1).

**Table 1:** Frequencies of positive predicting alleles.

Allele	Frequency (%)		*p*-value
	Controls(n = 714)	CD patients(n = 473)	
DQ2.5/DQ2.5	2.8	12.5	2.65E-11
DQ2.5/DQ2.2	2.5	23.6	5.09E-29
DQ2.5/DQ8	1.8	9.4	1.66E-08
DQ8/DQ2.2	2.5	1.5	9.27E-01
DQ8/DQ8	0.5	0.7	3.09E-01
DQ2.2/DQ2.2	1.5	0	N/A

The specificity derived from these CD risk genotypes achieved 92.9%, but sensitivity was only 45.5% (S1 Table, panel A). As demonstrated previously [14], using a combination of HLA and non-HLA variants can increase the diagnostic accuracy of genetic testing for CD. The aim of this study was, therefore, to find novel genetic variants associated with CD in the Polish population.

### Pooled-DNA allelotyping GWAS

A cost-effective GWAS was performed to search for new SNPs associated with CD in the Polish population. DNA sample pools from 336 CD patients and 264 controls were tested using Affymetrix Genome-Wide Human SNP 6.0. The allelotyping GWAS revealed 2 SNPs associated with CD with p <5*10^-8 (Table 2) in comparison to using the complete sample set. Both SNPs were accompanied by SNPs associated with CD with p-value<10^-5 positioned in close proximity (<150kb). When removing one control sample, the comparison confirmed both SNPs with slightly higher p-values (Table 2). No additional loci with more than one SNPs with p-value<10^-5 were discovered.

**Table 2:** GWAS-selected SNPs and associations with coeliac disease.

dbSNP RS ID	Chromosome	Chromosomal Position	*p*-value (complete dataset)	Adjusted *p*-value	*p*-value (high MAPD filtered)
**rs9272346**	**6**	**32604372**	**8.96E-09**	**0.0076**	**3.96E-07**
**rs3130484**	**6**	**31715882**	**1.68E-08**	**0.0076**	**2.30E-07**
rs3131379	6	31721033	4.23E-07	0.055	8.46E-07
rs2187668	6	32605884	5.81E-06	0.150	3.91E-05
rs9272723	6	32609427	8.77E-07	0.089	9.50E-07
rs3129716	6	32657436	4.12E-06	0.138	1.41E-05
rs9469246	6	32692227	3.04E-07	0.053	1.02E-05
rs9296042	6	32736005	4.16E-06	0.138	2.61E-06
rs9276689	6	32751962	3.21E-06	0.138	2.51E-05

**Fig 1:**
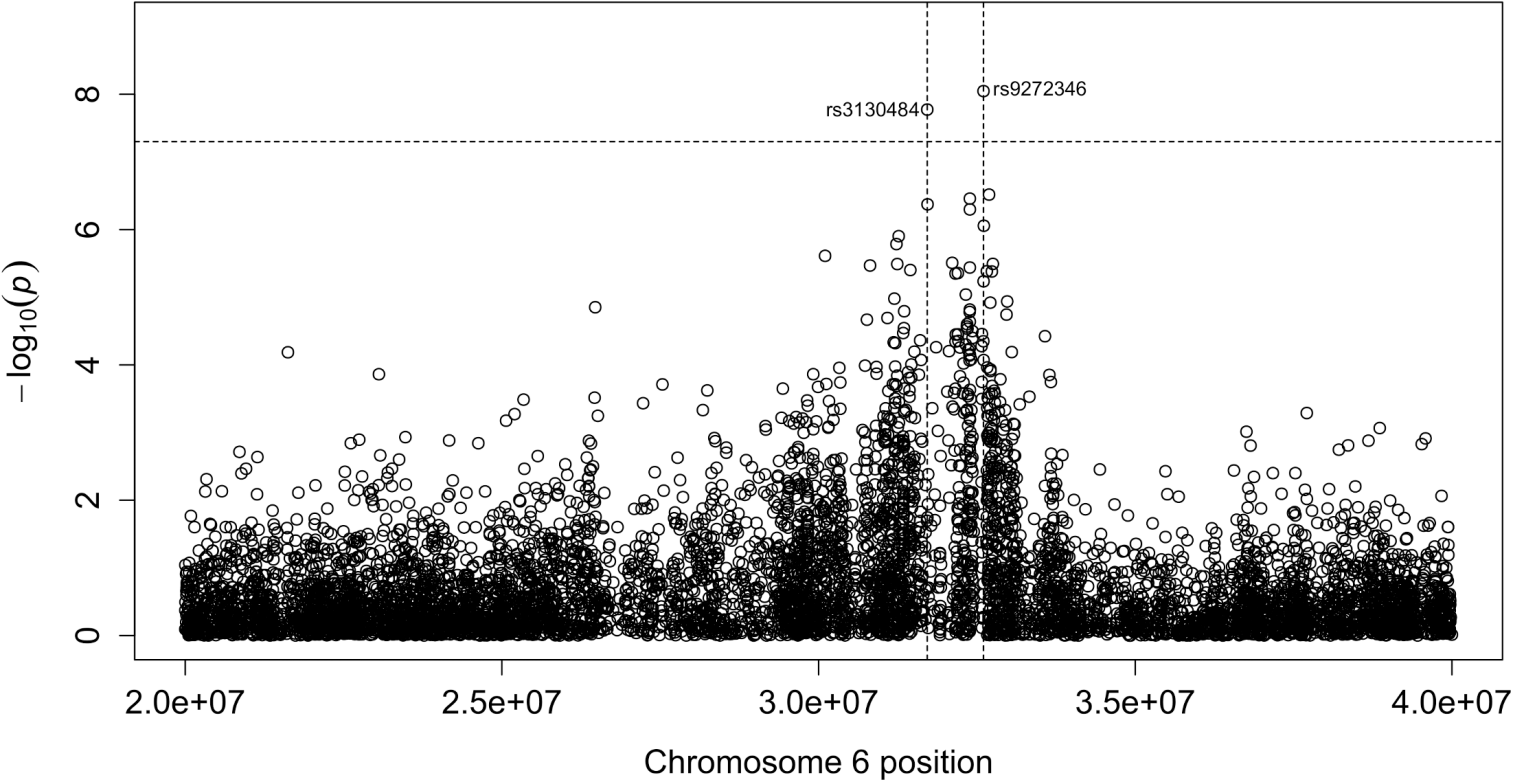
Manhattan plot showing CD associations with the susceptibility region on chromosome 6.

The two SNPs that associated most strongly with CD (p <5*10^-8) were rs3130484 (in *MSH5*) and rs9272346 (in *HLA-DQA1*) (Fig 1). These SNPs were validated by genotyping individual DNA samples from larger cohorts of 473 CD patients and 714 controls using TaqMan SNP Genotyping Assays. The difference in genotype frequencies of the two variants (Chi squared, p = 3.45E-69 and p = 1.24E-40, respectively) and the in allele frequencies between patient and control groups (p = 2.92E-64 and p = 2.19E-45, respectively) confirmed the association with increased CD susceptibility. The corresponding ORs (95% CIs) for the rs3130484 and rs9272346 variants were 6.10 (4.88–7.62) and 4.33 (3.51–5.35), respectively. Although the significance of CD association with both SNPs was lower when 357 healthy CD family members and 714 unrelated healthy controls were compared, highly significant associations were maintained: respective corrected p-values for rs3130484 (*MSH5*) and rs9272346 (*HLA-DQA1*) were 4.0E-18 and 3.8E-9 for genotype frequencies, and 2.5E-19 and 2.1E-10 for allele frequencies. The corresponding ORs (95% CIs) were 3.07 (2.39–3.94) and 1.89 (1.55–2.30). Thus, the rs3130484 and rs9272346 variants were significantly associated with CD patients and family members of CD patients. The rs9272346 SNP, although in strong linkage with HLA DQ2.5 loci, had only a moderately correlated genotype (S2 Table). Both SNP were also significantly associated with CD when considered simultaneously in conditional logistic regression model (S3 Table).

The MSH5 effect was independent from HLA with a p-value = 0.06/0.41 for the combinational term in logistic regression for dominant/recessive mode of inheritance.

Genetic diagnostic accuracy for CD was improved when the *HLA-DQ2*.*5* risk variants and the *MSH5* risk variant were considered in combination. Sensitivity increased to 74%, and specificity decreased slightly, to 80.3%. When the *MSH5* and *HLA-DQA1* risk variants were considered together, sensitivity and specificity were 68.9% and 82.8%, respectively.

## Discussion

The major histocompatibility (MHC) region on chromosome 6p21.3 contains more than 224 genes and is highly polymorphic. Risk variants within the MHC region are known to be associated with more than 100 different autoimmune and infectious diseases, including CD [18,19]. Approximately 95% of CD patients carry at least one of two risk haplotypes: DQ2.5 and DQ8 [14,18,20–23]. CD can also occur in patients with haplotype DQ2.2 and DQ7 encoded by respectively DQA1*0201/DQB1*0202 and DQA1*0505/DQB1*0301 [18]. However, these alleles are present at sufficient frequencies in the general population to suggest that their presence alone is insufficient for disease development.

To identify new genetic variants associated with CD in the Polish population, we processed pooled-DNA samples using microarrays to filter out uninformative genetic variance. 7 of 9 SNPs discovered using our methodology were found in the CD susceptibility region on chromosome 6p21.3, underlining the importance of the MHC region in CD pathogenesis. The two most significant SNPs (rs9272346 and rs3130484) were examined further in a larger cohort. One of these SNPs, rs9272346, was HLA-dependent (localized within 1 Kb of *DQA1*). The other SNP, rs3130484, was HLA-independent and mapped to *MSH5*, the protein product of which plays a crucial role in mismatch repair and meiotic homologous recombination [24]. *hMSH5* spans 25 Kb within the HLA region on chromosome 6p21.3 [25] and is associated with several coding region non-synonymous SNPs that are linked to human diseases including neoplasia, reproductive disorders, and several immune diseases [26]. Previous studies identified non-rs3130484 associations with several diseases including systemic lupus erythematosus, Kawasaki disease, DMT1, severe cutaneous adverse reactions to allopurinol, selective IgA deficiency, and common variable immune deficiency [26–28]. In turn, the rs9272346 variant is also associated with asthma [29] and DMT1 [30]. This study confirmed that multiple genetic variants within HLA and non-HLA genes from the HLA class III region may independently influence autoimmune disease susceptibility.

A previous study involving regular CD screening tests in Polish patients with DMT1 [31] showed that CD incidence in diabetic risk groups was about 6%. Undiagnosed CD cases were also found in 1/8 families that already contained a person with CD (Cukrowska B, personal communication). These observations highlight the importance of family-based and population-based screening for CD in at-risk groups to mitigate the long-term health problems associated with undiagnosed CD. Testing for HLA risk molecules is routinely performed using specialized kits, as described previously [18], but direct typing of HLA allelic variants is usually difficult and time-consuming due to their highly polymorphic structures that interfere with primer annealing.

Monsuur et al. [18] evaluated six tagging SNPs in CD patients and healthy control individuals for their ability to predict the *DQ2*.*2*, *DQ2*.*5*, *DQ7*, and *DQ8* alleles. Sensitivity, specificity, predictive value, and correlations between the SNP-based and HLA-based tests were assessed. This method was then used for cost-effective population screening to determine the prevalence of CD HLA risk alleles in different populations [14] where a 94%/54% sensitivity/specificity or 43%/93% sensitivity/specificity was achieved depending on the method version [14]. The present study used four of the six HLA-tagging SNPs [18] (rs7454108 for *DQ8*, rs7775228 and rs2395182 for *DQ2*.*2*, and rs2187668 for *DQ2*.*5*). The remaining two tagging SNPs, namely, rs4713586 and rs4639334, were withdrawn by the genotyping assay supplier. This withdrawal was probably due to interference of primer and probe annealing by neighboring variants. Our own attempts to design primer and Taqman probe pairs for these SNPs failed.

Six SNPs are needed to comprehensively predict the *DQ2*.*2*, *DQ2*.*5*, *DQ7*, and *DQ8* risk types. The four SNPs used in this study can correctly predict *DQ2*.*5*, *DQ8*, and *DQ2*.*2* (without excluding *DQ4*). Nevertheless, in this study, the positive prediction frequencies of *HLA-DQ2*.*5/DQ2*.*5*, *HLA-DQ2*.*5/DQ2*.*2*, and *HLA-DQ2*.*5/DQ8* were similar to those reported previously [14,18].

The negative predictive value of HLA genotypes reaches almost 100%. However, the risk of developing CD is not equal for all carriers of positive predicting alleles, and the positive predictive value is poor (~40%). HLA testing can be valuable in the identification of *HLA-DQ2* and/or *DQ8* carriers who can then be monitored by serological testing [14,18], but such serological tests are expensive and need to be repeated periodically. Genetic testing methods with improved sensitivity are, therefore, needed to more accurately identify individuals who would benefit from periodic antibody testing. Romanos et al. [14] demonstrated that the identification of potential CD patients was improved when 57 non-HLA variants were used alongside standard HLA tests. Combined use of the HLA and non-HLA risk variants decreased the positive predictive value from 94% to 57%, but sensitivity increased from 35% to 63%. This analysis reclassified 14.6% of the population into more accurate risk categories.

Our study found that CD genetic prediction was improved by examining both HLA and non-HLA SNPs. This corresponded with findings from previous studies. Our prediction matrix, which used four previously identified HLA-tagging SNPs and the *MSH5* SNP identified in this study, resulted in a decrease in specificity from 93% to 80% and an increase in sensitivity from 45% to 74% (when compared with prediction using the four HLA SNPs alone). When the two most significant SNPs identified in this study (*DQA1* and *MSH5*) were used in combination, specificity decreased to 82.8%, and sensitivity increased to 68.9%.

In summary, this study identified SNPs that improved prediction of CD risk. A diagnostic panel of predictive SNPs for clinical use may be developed from those uncovered in previous research and in this study.

## Supporting Information

S1 Fig QQ plot of p-values from comparison all CD samples vs all control samples (t-test). (TIFF)

S1 Table The performance of classifier based on HLA genotyping (panel A), MSH5 genotyping (panel B) and both: MSH5 and HLA genotyping (panel C). HLA DQ2.5/DQx "+" denotes any of genotypes found with increased frequency in CD patients: DQ2.5/DQ2.5, DQ2.5/DQ2.2 and DQ2.5/DQ8 (Table 1). HLA DQ2.5/DQx "-" denotes other genotypes.
(DOCX)

S2 Table The correlation between classical HLA typing and rs9272346 typing. HLA DQ2.5/DQx "+" denotes any of genotypes found with increased frequency in CD patients: DQ2.5/DQ2.5, DQ2.5/DQ2.2 and DQ2.5/DQ8 (Table 1). HLA DQ2.5/DQx "-" denotes other genotypes.
(DOCX)

S3 Table Results of conditional logistic regression for dominant and recessive mode of inheritance. (DOCX)
