# Supplementary figures and images for: Combination Testing Using a Single MSH5 Variant alongside HLA Haplotypes Improves the Sensitivity of Predicting Coeliac Disease Risk in the Polish Population
Source: PLoS One. 2015 Sep 25;10(9):e0139197. doi: 10.1371/journal.pone.0139197 (PMC4583383; doi:10.1371/journal.pone.0139197)

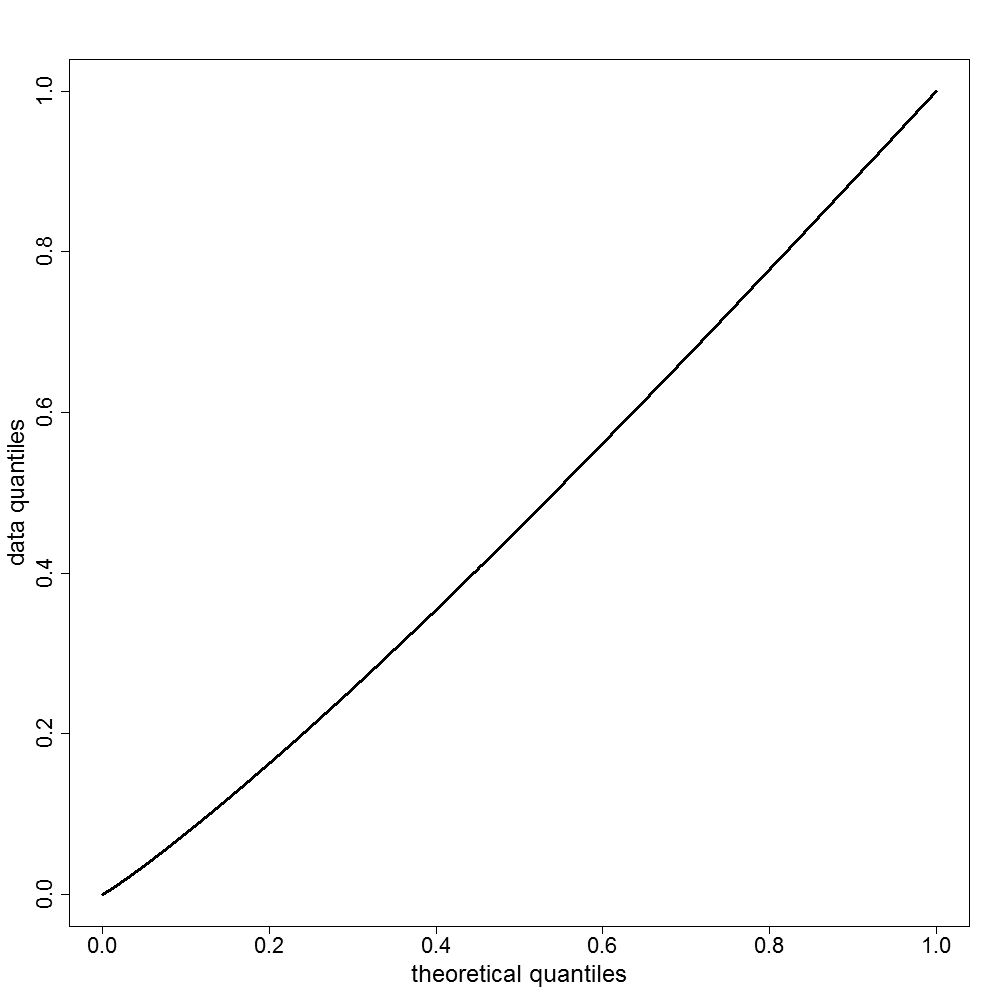

Supplement: S1 Fig — (TIFF) [file pone.0139197.s001.tiff]
