# Supplementary material for: Combination Testing Using a Single MSH5 Variant alongside HLA Haplotypes Improves the Sensitivity of Predicting Coeliac Disease Risk in the Polish Population
Source: PLoS One. 2015 Sep 25;10(9):e0139197. doi: 10.1371/journal.pone.0139197 (PMC4583383; doi:10.1371/journal.pone.0139197)
Supplement: S1 Table — HLA DQ2.5/DQx "+" denotes any of genotypes found with increased frequency in CD patients: DQ2.5/DQ2.5, DQ2.5/DQ2.2 and DQ2.5/DQ8 (Table 1). HLA DQ2.5/DQx "-" denotes other genotypes. (DOCX) [file pone.0139197.s002.docx]

**S1 Table.** The performance of classifier based on HLA genotyping (panel A), MSH5 genotyping (panel B) and both: MSH5 and HLA genotyping (panel C). HLA DQ2.5/DQx "+" denotes any of genotypes found with increased frequency in CD patients: DQ2.5/DQ2.5, DQ2.5/DQ2.2 and DQ2.5/DQ8 (Table 1). HLA DQ2.5/DQx "-" denotes other genotypes.

Yellow/green marking denotes true positive/true negative cases for each classifier.

**A.**

|  |  | **HLA DQ2.5/DQx genotype** | | | |
| --- | --- | --- | --- | --- | --- |
|  |  | **CD** | | **control** | |
|  |  | **-** | **+** | **-** | **+** |
| **rs3130484**  **(MSH5)** | **CC** | **119** | **27** | **560** | **17** |
|  | **C/T** | **135** | **144** | **85** | **24** |
|  | **TT** | **2** | **39** | **2** | **9** |
|  |  | **sensitivity** | **0.451** |  |  |
|  |  | **specificity** | **0.928** |  |  |

**B.**

|  |  | **HLA DQ2.5/DQx genotype** | | | |
| --- | --- | --- | --- | --- | --- |
|  |  | **CD** | | **control** | |
|  |  | **-** | **+** | **-** | **+** |
| **rs3130484**  **(MSH5)** | **CC** | **119** | **27** | **560** | **17** |
|  | **C/T** | **135** | **144** | **85** | **24** |
|  | **TT** | **2** | **39** | **2** | **9** |
|  |  | **sensitivity** | **0.687** |  |  |
|  |  | **specificity** | **0.828** |  |  |

**C.**

|  |  | **HLA DQ2.5/DQx genotype** | | | |
| --- | --- | --- | --- | --- | --- |
|  |  | **CD** | | **control** | |
|  |  | **-** | **+** | **-** | **+** |
| **rs3130484**  **(MSH5)** | **CC** | **119** | **27** | **560** | **17** |
|  | **C/T** | **135** | **144** | **85** | **24** |
|  | **(TT** | **2** | **39** | **2** | **9** |
|  |  | **sensitivity** | **0.745** |  |  |
|  |  | **specificity** | **0.803** |  |  |
