# Supplementary material for: Combination Testing Using a Single MSH5 Variant alongside HLA Haplotypes Improves the Sensitivity of Predicting Coeliac Disease Risk in the Polish Population
Source: PLoS One. 2015 Sep 25;10(9):e0139197. doi: 10.1371/journal.pone.0139197 (PMC4583383; doi:10.1371/journal.pone.0139197)
Supplement: S2 Table — HLA DQ2.5/DQx "+" denotes any of genotypes found with increased frequency in CD patients: DQ2.5/DQ2.5, DQ2.5/DQ2.2 and DQ2.5/DQ8 (Table 1). HLA DQ2.5/DQx "-" denotes other genotypes. (DOCX) [file pone.0139197.s003.docx]

**S2 Table.** The correlation between classical HLA typing and rs9272346 typing. HLA DQ2.5/DQx "+" denotes any of genotypes found with increased frequency in CD patients: DQ2.5/DQ2.5, DQ2.5/DQ2.2 and DQ2.5/DQ8 (Table 1). HLA DQ2.5/DQx "-" denotes other genotypes.

|  |  | HLA DQ2.5/DQx genotype | | | |
| --- | --- | --- | --- | --- | --- |
|  |  | CD | | control | |
|  |  | - | + | - | + |
| rs9272346 | AA | 5 | 0 | 129 | 5 |
|  | A/G | 123 | 3 | 311 | 8 |
|  | GG | 121 | 207 | 196 | 34 |
