# Supplementary material for: Combination Testing Using a Single MSH5 Variant alongside HLA Haplotypes Improves the Sensitivity of Predicting Coeliac Disease Risk in the Polish Population
Source: PLoS One. 2015 Sep 25;10(9):e0139197. doi: 10.1371/journal.pone.0139197 (PMC4583383; doi:10.1371/journal.pone.0139197)
Supplement: S3 Table — (DOCX) [file pone.0139197.s004.docx]

**S3 Table.** Results of conditional logistic regression for dominant and recessive mode of inheritance.

|  | p-value | OR |
| --- | --- | --- |
| dominant | | |
| rs31304841 | 9.68E-14 | 2.37 (1.89-2.97) |
| rs92723461 | 3.37E-05 | 8.19 (3.03-22.1) |
| DQ2.5 | 2.39E-06 | 1.66 (1.34-2.04) |
| recessive | | |
| rs31304841 | 0.92 | 1.02 (0.72-1.42) |
| rs92723461 | 8.63E-07 | 1.81 (1.43-2.30) |
| DQ2.5 | 3.90E-11 | 2.12 (1.69-2.64) |
